# Origin of the Photoluminescence Quantum Yields Enhanced by Alkane-Termination of Freestanding Silicon Nanocrystals: Temperature-Dependence of Optical Properties

**DOI:** 10.1038/srep36951

**Published:** 2016-11-10

**Authors:** Batu Ghosh, Masaki Takeguchi, Jin Nakamura, Yoshihiro Nemoto, Takumi Hamaoka, Sourov Chandra, Naoto Shirahata

**Affiliations:** 1 International Center for Materials Nanoarchitectonics (MANA), National Institute for Materials Science (NIMS), 1-1 Namiki, Tsukuba, Ibaraki 305-0044, Japan; 2 Department of Physics, Triveni Devi Bhalotia College, Raniganj, Burdwan-713347, West Bengal, India; 3 Transmission Electron Microscopy Station, NIMS, 1-2-1, Sengen, Tsukuba, Ibaraki 305-0047, Japan; 4 PRESTO, Japan Science and Technology Agency (JST), 4-1-8 Honcho Kawaguchi, Saitama 332-0012, Japan

## Abstract

On the basis of the systematic study on temperature dependence of photoluminescence (PL) properties along with relaxation dynamics we revise a long-accepted mechanism for enhancing absolute PL quantum yields (QYs) of freestanding silicon nanocrystals (ncSi). A hydrogen-terminated ncSi (ncSi:H) of 2.1 nm was prepared by thermal disproportination of (HSiO_1.5_)_*n*_, followed by hydrofluoric etching. Room-temperature PL QY of the ncSi:H increased twentyfold only by hydrosilylation of 1-octadecene (ncSi-OD). A combination of PL spectroscopic measurement from cryogenic to room temperature with structural characterization allows us to link the enhanced PL QYs with the notable difference in surface structure between the ncSi:H and the ncSi-OD. The hydride-terminated surface suffers from the presence of a large amount of nonradiative relaxation channels whereas the passivation with alkyl monolayers suppresses the creation of the nonradiative relaxation channels to yield the high PL QY.

Intensive studies are ongoing into freestanding silicon nanocrystals (ncSi), which is a promising material for applications including light emitting diodes (LEDs)^1^,^2^,^3^, optically-pumped lasers^4^, optical switching devices^5^, sensors^6^,^7^, hydrogen storage^8^, field-effect transistors^9^, solar photovoltaics^10^,^11^, medical and biological applications^12^,^13^,^14^,^15^, photodiodes^16^, and lithium-ion batteries^17^. The appeal of ncSi in these applications is largely a result of its exceptional properties of nontoxicity^18^, continuous tunability of photoluminescence (PL) over a broad spectral range from UV to near-IR^19^, third-order nonlinear optical behavior^20^,^21^, robustness of the covalent Si-Si bond versus ionic bonds, high compatibility with microelectronics, and being an abundant element. Since the discovery of porous Si in 1990^22^, the PL properties of ncSi have been extensively investigated. A long-running debate continues over the origin of PL which might be characteristic of the indirect bandgap nature. The indirect bandgap structure could be inherited even in such a small crystal, but the size-dependent PL properties suggest the appearance of quantum confinement (QC) in nanocrystals (NCs) smaller than 5 nm^23^,^24^,^25^. Nevertheless, the absolute PL quantum yields (QYs) of hydrogen-terminated Si NCs (ncSi:H) that serve as a fundamental surface for microfabrication are still low (<10%)^26^,^27^,^28^. The ncSi-based light emitters still have shown poorer optical performances compared to the devices with freestanding core/shell quantum dots (QDs) of direct bandgap compound semiconductors.

A general approach to enhancing the PL QYs of QDs includes the removal of surface defects/traps that cause nonradiative recombination of photogenerated electron-hole (*e-h*) carriers. Chemically modifying of their surface is an efficient approach for this purpose. For ncSi:H, a well-developed surface chemistry employs molecules that have terminal unsaturated bonds, such as 1-alkenes or 1-alkynes. These are used for hydrosilylation, which was first performed on bulk Si. The covalent attachment of hydrocarbon chains onto the surface of ncSi began with the pioneering work of Kauzlarich *et al*. to allow NCs to be dispersed as colloids in nonpolar solvents including toluene^29^. Another merit offered by the hydrosilylation compared to other techniques is the incredible enhancement of PL QYs^1^,^2^,^12^,^15^,^30^. Much effort has been devoted to increasing PL QYs by different synthesis methods in different laboratories, and high PL QYs (>40%) have been achieved thanks to termination with alkyl monolayers^15^,^31^,^32^. It is commonly believed that the passivation of ncSi surface with a monolayer (similar to other compositions including CdSe) reduces surface defects/traps that serve as nonradiative recombination centers. However, this long-accepted mechanism does not convincingly explain the low PL QYs of ncSi:H. The fact remains that termination with hydrogen atoms is supposed to remove the defects/traps at the NC surface^23^. Dohnalová *et al*. reported that the termination of ncSi with alkyl monolayers leads to a dramatic modification of the energy structure of ncSi to give a phonon-less character for radiative excitonic recombination close to the band edge, yielding an enhanced PL QY^30^. Hannah *et al*. explored the pressure-dependent PL properties of alkyl-terminated ncSi, and drew the conclusion that the emission originates from the X-to-Γ transition featured in the bulk geometry. This suggests a role for surface ligands that lack electronic density of states (DOS) within the energy gap^33^. In order to obtain insight regarding further enhancement of PL QYs, it is necessary to determine the extent to which the nonradiative recombination is suppressed by the alkyl monolayers in contrast to the passivation of hydrogen atoms.

In this study, we systematically investigated the temperature dependence of PL properties with relaxation dynamics of the freestanding ncSi with different terminal groups to revise the long-accepted mechanism concerning the role of surface monolayers.

## Results and Discussion

### Synthesis and characterization

Two sets of freestanding NCs were prepared: ncSi:H and ncSi terminated with octadecane monolayers (ncSi-OD) (see Methods). In brief, triethoxysilane (TES) was employed as a starting precursor. The hydrolysis product, i.e., (HSiO_1.5_)_*n*_, of TES was disproportionated at 1100 °C into two different oxidation states Si^0^ and Si^4+^ according to Eqs (1) and (2). The oxidation states at each step were determined in our previous study, in which X-ray photoelectron spectroscopy (XPS) was used for clarification^1^. After cooling to room temperature, the dark-brown solid (i.e., oxide-embedded ncSi) was mechanically ground in an agate mortar with a pestle. The fine powder thus obtained was stirred in a mixture of ethanol and 48% HF_(aq)_ to liberate ncSi:H from the oxide. Thermal hydrosilylation was carried out at 170 °C in a 1:1 volumetric-ratio mixture of mesitylene/1-octadecene to yield the ncSi-OD.


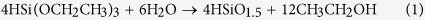



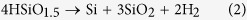


**Figure 1:**
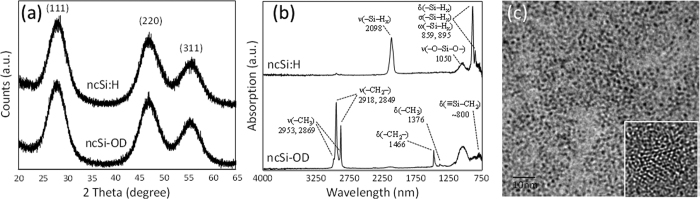
Summary of the typical results for ncSi:H and ncSi-OD characterized by (**a**) XRD, (**b**) ATR-FTIR, and (**c**) HR-TEM. The HR-TEM image was obtained from ncSi:H. The inset is a typical photograph of 2.1-nm ncSi:H. The estimated full width at half maxima (FWHM) of the diffraction peaks of (111), (220), and (311) planes were 4.43 ± 0.025°, 4.99 ± 0.04° and 5.32 ± 0.06°, respectively, for ncSi:H and 4.35 ± 0.02°, 4.73 ± 0.03° and 5.20 ± 0.05°, respectively, for ncSi-OD.

Typical X-ray powder diffraction (XRD) patterns and attenuated total-reflectance Fourier-transform infrared (ATR-FTIR) spectra of the ncSi:H and the ncSi-OD are presented in Fig. 1a,b, respectively. The XRD patterns index to a diamond cubic lattice of Si, with significant peak broadening. The broadened powder-diffraction peaks are caused by the small size of the NCs and the inhomogeneous strain of the crystalline lattice according to the well-established Williamson-Hall theory. Interestingly, the diffraction lines of the ncSi-OD became slightly narrower than those of the ncSi:H despite both samples being prepared from the same oxide-embedded ncSi (see the caption for Fig. 1a). This suggests that the diamond cubic lattice of ncSi:H was distorted more than that of ncSi-OD. Figure 1c shows a representative high-resolution transmission electron microscopy (HR-TEM) photograph taken from the spherically shaped ncSi:H that is ~2.1-nm in diameter. The inset is a typical high-resolution image of 2.1-nm ncSi:H. The contrasted lattice image that corresponds with the diamond cubic Si structure is consistent with the XRD pattern, but we cannot clarify the surface structure of the ncSi:H from the image. A typical ATR–FTIR spectrum of the ncSi:H exhibits a prominent feature centered at ~2098 cm^−1^, and a doublet in the range 910–845 cm^−1^. The region near 2098 cm^−1^ is attributed to the SiH_x_ stretching mode^34^. The doublet is composed of two sets of absorption peaks. The first set centered at 895 cm^−1^ is attributed to the SiH_3_ degenerate deformation and SiH_2_ scissoring modes, while the second set centered at 859 cm^−1^ is assigned to the SiH_3_ symmetric deformation and SiH_2_ wagging modes^35^. All these bands are significantly diminished by the hydrosilylation, which instead exhibits characteristic C–H stretching and bending/scissoring bands at 2850–2960 and 1350–1500 cm^−1 ^36. An absorption peak appears at ~780 cm^−1^ because of the scissoring Si–CH_2_ vibration. The presence of a small amount of surface oxide is evidenced by O–Si–O stretching at 1030 cm^−1^.

### Temperature dependence of PL properties

**Figure 2:**
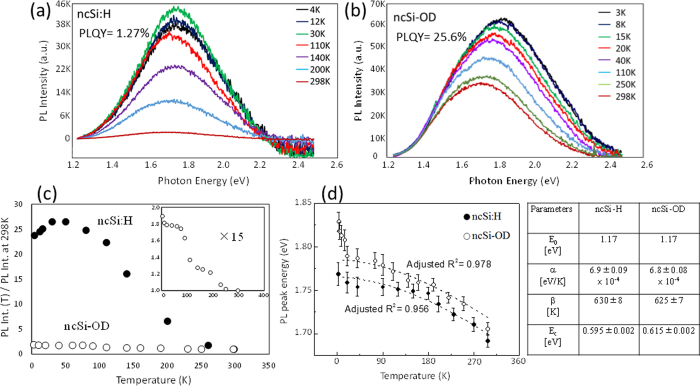
PL spectra as a function of temperature in the range 3–298 K for (**a**) ncSi:H and (**b**) ncSi-OD. (**c**) Integrated PL intensity of ncSi:H and ncSi-OD as a function of temperature. All the values of PL intensity were normalized to the values recorded at 298 K. The temperature-dependent evolution for ncSi-OD was magnified 15 times (inset). (**d**) Temperature-dependent PL peak energies for ncSi:H and ncSi-OD. The estimated parameters (i.e., α, β and Ec) for ncSi:H and ncSi-OD are listed in the table.

It is established that hydrosilylation of 1-alkenes or 1-alkynes increases PL QYs. In this work, absolute PL QYs were estimated by a standardized integrating-sphere method. The samples -in solid form as powders placed in short column-shaped cuvettes (*Φ*20 mm) of quartz glass- were used for the measurement of their PL QYs. The estimated PL QY for the ncSi:H was 1.27% at maximum, whereas the value for the ncSi-OD was 25.6% at the same excitation. We investigated the PL properties as a function of temperature in order to discuss the origin of this anomalous enhancement of the PL QY. In this work, two quartz glass substrates covered with thin films of ncSi:H and ncSi-OD, respectively, were used as samples. The PL spectra of the ncSi:H and ncSi-OD were measured in the range 3–298 K with a strict temperature fluctuation control of ±0.1 K. The samples were cooled down to 3 K and heated back to 298 K without any apparent damage to the optical properties, including PL spectral shape, intensity, position, and QY. Figure 2a,b show the temperature dependence of the PL spectra excited at 375 nm for ncSi:H and ncSi-OD. An increase in temperature results in the redshift of the PL spectra, along with spectral broadening. The obtained dependencies are in agreement with those of QDs of other semiconductors^37^,^38^. However, we see a large difference in the evolution of integrated PL intensity between ncSi:H and ncSi-OD. Figure 2c shows the plots of PL intensity at each temperature normalized with respect to the value at 298 K. For ncSi:H, the PL intensity increases with a rise in temperature from 3 to 30 K, but starts to decrease at ~30 K. Afterward, the PL intensity continues to decline up to 298 K. The dependence in the range 3–30 K is in direct contrast to the well-known trends of QDs of other semiconductors. A 13-fold reduction in PL intensity is observed as the temperature increases from 3 to 298 K. Such a dramatic decrease in PL intensity indicates the presence of a large amount of nonradiative relaxation channels even at the ncSi surface, which is supposed to be terminated with hydrogen atoms in high density. Such a large amount of nonradiative channels causes a strong thermal quenching. These unexpected evolutions will be discussed later taking into account the radiative and nonradiative decay times. For ncSi-OD, the PL intensity normalized with respect to the value at 298 K steadily decreases over the whole temperature range, unlike the case for nSi:H. Surprisingly, in the temperature range 3–298 K, the PL intensity shows a minimal decrease of 45%, which means a weak thermal quenching. This suggests that the nonradiative relaxation process is remarkably suppressed in the interior of the NCs terminated with alkyl monolayers.

The temperature dependence of bandgaps in QDs of semiconductors has been extensively studied to discuss their PL origin (whether they arise from the QC effect or from highly localized defect states at the surface)^37^,^38^. In Fig. 2a,b, the PL spectra shift toward the lower photon energy side with increasing temperature for both samples. The measured values of the peak emission energy are plotted versus temperature in Fig. 2d. On the assumption that the redshift of PL spectra reflects the shrinkage of the bandgap of ncSi as well as the QDs of other semiconductors, the variation of PL peak energy appears due to lattice expansion and electron-phonon coupling with increasing temperature. Thus, the temperature coefficient of the bandgap, d*E*_g_/d*T* can be expressed as^39^,^40^


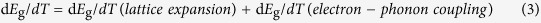


For semiconductors, due to the temperature dependence of lattice parameter, *a*, with [*∂E/∂T*]_lattice_ = [*∂E/∂a*] [*∂a/∂T*], the lattice expansion generally affects E(*T*). However, because of the magnitude of [*∂E/∂a*], the evolution of the bandgap as a function of temperature is influenced more by the electron-phonon interaction than by lattice deformation. Since the photogenerated *e-h* carriers are spatially confined in a small box of NC, the electron-phonon interaction is strengthened, resulting in a larger value of d*E*_g_/d*T* than the values for bulk crystals of semiconductors^41^. Furthermore, the crystalline lattice of the NC is possibly distorted more than that of a bulk crystal^42^. Therefore, the PL peak energy, E(*T*), is a decreasing function of the temperature. It is well known that the temperature dependence of PL spectra of QDs of semiconductors follows the empirical Varshni relation^43^. For ncSi, all the published work, to the best of our knowledge, has used the ncSi-embedded SiO_x_ films instead of freestanding ncSi, and has reported that the temperature dependence of the bandgap energy in the range from cryogenic to room temperature could not be reproduced by either the relation or its modified one in contrast to the cases of QDs of other semiconductors^44^,^45^. This conflict complicates a better understanding of the origin of PL for ncSi. We resolve this difficulty for the first time by using a freestanding red-light-emitting ncSi:H as a model. Dohnalová *et al*. reported the temperature dependence of PL properties of blue-light-emitting Si QDs^46^, but our work gives a result that substantially differs from their conclusion as discussed later. For fitting, we employed the empirical Varshni relation modified with an extra corrected term for the QC energy as expressed by^45^


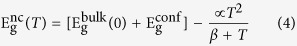


where α is the temperature coefficient (d*E*_g_*/*d*T*), β is close to the Debye temperature, 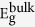
(0) represents the bulk bandgap of Si obtained at 0 K ( = 1.17 eV), and 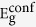 is a value of the QC energy corresponding to the difference in energy between the PL peak and bulk bandgap at 298 K. From the Eq. (4), it is clear that the relation is quadratic with temperature at low temperatures, and becomes linear at higher temperatures. In our experiment, the highest observed temperature is 300 K, which is appreciably lower than the value of β. For lower temperatures, it is not possible to unambiguously estimate the values of the parameters α and β separately from the fitted curve since, in the region T ≪ β, the curve is more dependent on the ratio of α, and β. As the temperature approaches room temperature, it becomes more feasible to estimate α and β more accurately from the fitted curve with a narrow uncertainty. However, we fitted the experimental data with the equation over the whole experimental temperature range (3–300 K) with adjusted R^2^ values greater than 0.956 and 0.978 for ncSi-H and ncSi-OD, respectively, which supports good fits. The estimated values of α, β, and 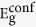 are tabulated in Fig. 2d. As depicted by the fitted curve (black dotted-line), the measured plots for ncSi:H could be reproduced by Eq. (4), indicating that the dominant emission in the NCs arises from the radiative *e-h* recombination based on the QC effect^37^,^47^. For ncSi-OD, the PL peak energy agrees very well with the equation over the range 20–298 K. However, we see a strong discrepancy to this relation in the temperature range 3–20 K. The value of *α* for ncSi:H, being almost equal to that for ncSi-OD, is more than double the value for bulk Si (*α* = 0.317 meV/K). A theoretical study predicts an increase of the coefficient α with a reduction in the diameter of ncSi^47^. Experimental work by Derr, *et al*. corroborates the prediction with an estimated value of α = 0.5 meV/K for 2.0 nm of ncSi embedded in an Si-rich oxide (SRO) film^48^. The coefficient *α* estimated in our work is larger than the reported values^45^,^48^. The estimated values of the parameter *β* are close to the Debye temperature for bulk Si (640 K). The values of the confinement energy (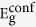) are ~0.3 eV larger than those of the ncSi embedded in SiO_2_ films^45^,^49^. These results suggest that the confinement of photogenerated *e-h* carriers is strengthened in the freestanding NCs terminated with hydrogen atoms or alkyl monolayers, in contrast to the case of the ncSi embedded in oxide films.

### Temperature dependence of PL decay dynamics

The evolution of the bandgap energy with temperature implies that the confinement of photoexcited carriers in ncSi-OD are strengthened than that in ncSi:H. The rapid decline of the PL intensity with increasing temperature observed for ncSi:H indicates the presence of a large number of nonradiative channels. With increasing temperature, these nonradiative channels become thermally activated. The PL QY, η, can be expressed as η = k_r_/(k_r_ + k_nr_), where k_r_ = 1/τ_r_ and k_nr_ = 1/τ_nr_ (τ is the characteristic PL lifetime). In this context, we next investigated the temperature dependence of the decay dynamics for ncSi:H and ncSi-OD to gain a better understanding of the relaxation and recombination processes of the carriers in the NCs with different terminations. Dohnalová, *et al*. reported a theoretical study of the change in radiation rate and recombination process due to the attachment of alkyl monolayers, which switches ncSi from an indirect-bandgap-like material to a direct-one^50^. In our study, we could not obtain any experimental evidence that suggested such a dramatic change in the *e-h* recombination process. The measured PL properties implied the indirect bandgap nature that was inherited even in nanostructured Si with hydrocarbon chains, as discussed below.

In general, the relaxation processes in QDs of semiconductors include radiative relaxation, Auger nonradiative scattering, Förster energy transfer between QDs with different physical sizes and/or shapes, thermal escape or tunneling, and thermally activated trapping at surface defects/traps states. Auger recombination becomes a significant parameter under non-equilibrium conditions or only when carrier density of incident light is very high. This is possible if the excitation energy is very high. In our experimental regime, the average number of carriers excited by irradiation of the LED is, however, small enough to neglect Auger nonradiative recombination. As is mentioned above, the large Stokes shift between absorption and emission prevents the Förster energy transfer between NCs. Thus, the temperature dependence of PL decay time (τ) can be expressed by^39^


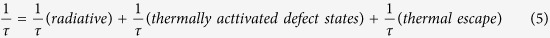


**Figure 3:**
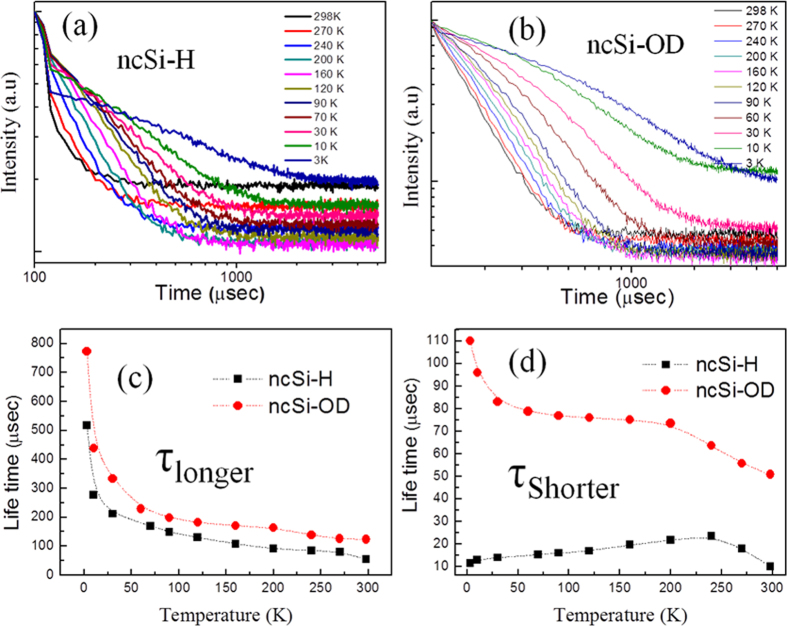
PL decay profiles for (**a**) ncSi:H and (**b**) ncSi-OD, measured over the temperature range 3–298 K. Temperature dependence of the characteristic PL decay time: (**c**) longer component and (**d**) shorter component for ncSi:H and ncSi-OD, respectively.

Figure 3a,b show the PL decay curves, measured at the maxima of PL intensity, as a function of temperature for ncSi:H and ncSi-OD. The curves were fitted with a bi-exponential function, suggesting two independent processes for the bi-exponential decay fitting. Figure 3c,d show the estimated values of the fast and slow components of PL lifetime as a function of observation temperature. In Fig. 3c, we see a similar evolution between ncSi:H and ncSi-OD. The values of the longer PL lifetime (i.e., slow component) decrease with a rise in temperature. This implies an increased opportunity for recombination dominated by nonradiative processes, since their channels become active as the temperature increases. Therefore, the decay time could be described by a decreasing function of temperature. Interestingly, this observation gives a result that is opposite to the previous claim involving the temperature dependence of PL decay time at nanosecond scale for the blue luminescent Si QDs used as a sample^46^. This discrepancy might be due to the PL origin of the red-light-emitting ncSi that differs from that of the blue-emitting ncSi.

In Fig. 2c, we see a monotonic decrease in the normalized PL intensity with increasing temperature for ncSi-OD. In contrast, we see a discrepancy in the evolution of the normalized PL intensity between ncSi:H and ncSi-OD. Below 30 K, the normalized PL intensity of ncSi:H slightly increases with a rise in temperature in spite of the rapid decrease of PL decay time. Thus, it is clear that the decay time decrement in the range of 3–30 K cannot be explained based only on the nonradiative recombination mechanism. The fact that the values of PL decay time at low temperatures are larger than those for ncSi:H is perhaps a result of the increase of radiative decay time. It could be explained by taking account of the thermal equilibrium of PL lifetime that was proposed by Calcott *et al*.^51^. According to that model, the ground state, split by energy ΔE, gives rise to an upper singlet excitonic state (spin allowed) and a lower triplet state (spin forbidden) because of the exchange interaction of electrons and holes. The value of ΔE for bulk Si is usually very small (~1 meV) at room temperature (*k*_B_T = ~26 meV, where *k*_B_ is the Boltzmann constant). In contrast, the QC effect generated in ncSi smaller than 5 nm enhances the spin-exchange interaction, causing a large ΔE. For ncSi of 2 nm, the reported values of ΔE are 8–20 meV^52^, which are comparable to the thermal energy at room temperature in some cases. Therefore, the triplet state must be populated at temperatures below 30 K because these two states are separated by a large splitting energy. In such a situation, an optical transition from the triplet state is ideally forbidden, but is slightly allowed in practice because of the spin-orbit interaction, leading to a long radiative lifetime (~1 ms)^45^.

The overall trend in the variation of longer lifetime with temperature for ncSi-OD is quite similar to that for ncSi:H. The values are down to 55 μs and 123 μs at 298 K for ncSi:H and ncSi-OD, respectively. A PL lifetime of a few multiple of 10 μs is a characteristic that is common to all Si:H NCs that emit light in the orange-red range^19^. At 298 K, the surface termination with hydrocarbon chains raises the decay time to 123 μs. According to the scenario outlined above, this is possibly due to the suppression of nonradiative pathways by the hydrocarbon chains that are covalently bonded to the ncSi surface. For ncSi:H, the significant decrease in the longer component from 510 to 55 μs over the whole temperature range is because of thermal activation of nonradiative channels. For ncSi-OD, a dramatic decrease in decay time from 791 to 230 μs occurs in the temperature range 3–65 K. In addition, a slow decrease of the decay time from 230 to 123 μs is seen in the range 65–298 K. This slow decrease might be due to the presence of the surface monolayers that lessen the contribution of nonradiative decay time to the total PL lifetime, which is given by Eq. (5), in contrast to the case for ncSi:H.

In Fig. 3d, the temperature dependence of the short component of PL lifetime shows a significant difference between ncSi-H and ncSi-OD. The fast-decay-components values at 298 K were 17 and 50 μs for ncSi-H and ncSi-OD, respectively. For ncSi-OD, the decay time decreased slower as the temperature increased when compared to the case of the longer component. In contrast, for ncSi:H, the decay time continued to slowly increase with temperature up to 240 K. This type of behavior, which is not as common in the temperature dependence of PL lifetime has not yet been reported for ncSi. However, similar trends have been reported for NC systems of other semiconductors^53^,^54^. According to the literature, this behavior could be explained by nonradiative mechanisms such as a depopulation process from trap states to exciton states or a phonon/defect scattering process. The time-resolved PL study also suggests the presence of nonradiative channels that are preferentially created in the nanostructure of ncSi:H.

### Possible mechanism for PL QY enhanced by hydrosilylation

A question arises as to why the appreciable increase in the PL QY as the NC surface ligands are replaced with hydrocarbon chains from hydrogen atoms. A detailed analysis of the temperature dependence of PL intensity for ncSi has been performed using ncSi embedded into SiO_2_^45^ and ncSi/SRO^55^. Those previous studies used oxide-embedded NCs rather than freestanding NCs, and concluded that both defects and traps generated at the interface between ncSi and oxide serve as nonradiative channels. Such inhomogeneous interfaces are invoked to explain the mechanism of evolution of PL properties as a function of temperature. However, the results of our work show that this long-accepted origin alone is not enough to deduce the mechanism of the unexpected enhancement in PL QY that arises from the hydrosilylation. Indeed, a comparison of the temperature dependence of PL properties between ncSi:H and ncSi-OD indicates the presence of a larger number of nonradiative channels in ncSi:H than in ncSi-OD. One common nonradiative channel is definitely the defect/trap state formed at the interface between the surface oxide and ncSi for both samples (see the ATR-FTIR spectra). Although it is difficult to make a quantitative comparison in the amount of surface oxide between ncSi:H and ncSi-OD, the surface of ncSi-OD would be expected to contain far higher levels of oxide than that of ncSi:H because of a lower packing density of alkyl monolayers relative to hydrogen atoms^56^. Nevertheless, we see the rapid decrease of PL intensity with temperature for ncSi:H rather than for ncSi-OD. This implies the presence of other nonradiative channels in the interior of ncSi:H.

**Figure 4:**
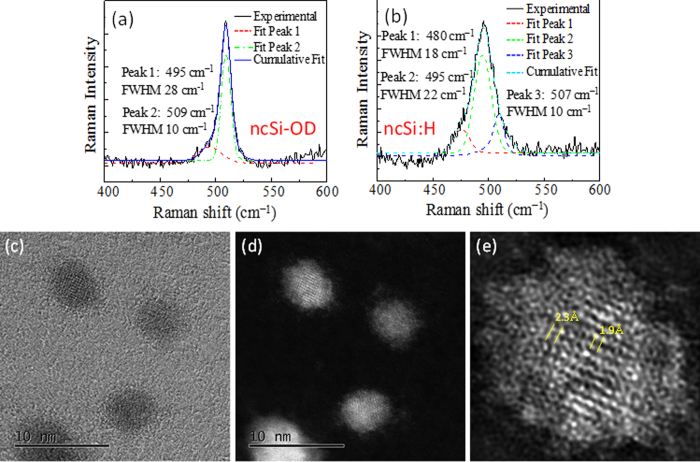
Raman scattering spectra of (**a**) ncSi-OD and (**b**) ncSi:H. Graphs (**a,b**) show the fitting of amorphous, intermediate (or under-coordinated) atomic configurations, and crystalline components of ncSi-OD and ncSi:H, respectively. (**c–e**) STEM images of ncSi:H. From left to right: (**c**) ABF-, (**d**) HAADF-, and (**e**) HAADF-STEM images. (**e**) A magnified image of the NC positioned at the top-left of the image (**d**). A low-pass filter was applied to acquire the image (**e**).

To visualize experimentally the nonradiative channels proposed in our work, we used a combination of Raman spectroscopy and scanning TEM (STEM). Figure 4a,b show the typical Raman scattering spectra of ncSi-OD and ncSi:H. These were recorded in the *Φ*10-μm focused area of a 532-nm laser with a power density of 9 kW/cm^2^, which was too low to influence the Raman spectral shape and position^57^. In a bulk crystal of Si, the optical phonon is a Raman active mode because of its zero momentum, and occurs at the center of the Brillouin zone (q = 0) to give a sharp peak at a Raman shift of 521 cm^−1^. In contrast, crystal momentum is no longer conserved in a single nanoscale of ncSi, particularly for sizes smaller than 6 nm^57^. In such a small NC, more phonons at q ≠ q_0_ (away from the center of the Brillouin zone) can contribute to the phonon DOS, leading to the shift of the peak to lower wavenumbers and the spectral broadening of the transverse phonon (TO) line^57^,^58^. In Fig. 4a, the Raman peak of ncSi-OD shifts predictably toward the lower frequencies, and is dominated by a TO peak centered at 509 cm^−1^, along with a low-frequency shoulder at 495 cm^−1^. The spectrum was not fitted with a Fano line shape^59^. According to the literature, a Raman shift of 509 cm^−1^ arises from the ncSi of ~2.1 nm^58^,^60^. The additional vibrational mode is seen at lower frequencies in the range 490–497 cm^−1^. This noteworthy frequency redshift with respect to bulk Si, which is frequently found in visible-light-emitting Si NCs, has been reported by various laboratories where different methods are employed for ncSi preparation. The clarification of its origin is a challenging subject, but several possible mechanisms have been suggested. Kůsová *et al*. point to the tensile strain that is caused by lattice distortion of diamond cubic Si, leading to a modified Raman signal as a result of phonon energy influenced by the strain^61^. Some other theoretical and experimental studies invoke distortion of the crystalline lattice, such as an intermediate range between the long-range order of the crystalline state and the short-range order of the amorphous state^58^,^60^,^62^,^63^,^64^. It is predicted that the lattice distortion occurs at the surface of ncSi rather than at its center. One of the theoretical studies suggests that under-coordinated surface atoms form a shorter Si-Si bond than that of bulk Si^60^. HR-TEM observation provides direct evidence of the shortened Si-Si bond lengths in 2.4-nm ncSi^65^. The bond length between atoms decreases with size reduction^65^. As suggested in those studies, the surface reconstruction might be understood to be caused by a high surface-area-to-volume ratio at the size scale discussed in this work. Our Raman spectroscopic study verifies that ncSi-OD could be dominated by a structurally coherent diamond cubic lattice, along with the subtle under-coordinated configuration at the surface. The true atomic arrangement of the surface remains a matter of debate: a similar argument has also been made for nanocrystalline germanium (ncGe)^42^,^66^. In contrast, the Raman spectrum of ncSi:H exhibits a main peak at 495 cm^−1^ (see Fig. 4b). The small contributed peak at 480 cm^−1^ arises from amorphous Si (a-Si)^63^. The observation of vibrational SiH_3_ and SiH_2_ modes in the ATR-FTIR spectrum suggests the presence of a-Si in the persistent shell of ncSi:H. A theoretical study by Pizzagalli *et al*. discusses a stable NC structure of 1–2.5 nm diameter using Ge as a model^42^. According to their calculation, the stable ncGe is mainly composed of a crystalline core (i.e., diamond Ge), but its surface is reconstructed at the atomic scale to minimize the surface free energy. The reconstructed surface no longer exhibits bulk-like geometry, but instead an intermediate structural configuration. A similar suggestion is reported by Sapelkin *et al*., who revealed that the presence of a thick distorted surface layer kinetically stabilizes 5-nm ncGe^66^. These investigations support an argument for a crystalline lattice near the surface region of ncSi that is structurally distorted to form an amorphous phase, whereas the core is composed of diamond cubic Si.

The discussed ncSi:H nanostructure is experimentally underpinned by annular bright-field (ABF) STEM images that were simultaneously recorded with high-angle annular dark-field (HAADF) images. Figure 4c,d show representative ABF and HAADF STEM images, respectively, of ncSi:H. These images provide complementary information. The dark spots in the ABF image represent atomic columns of Si. Because of the high sensitivity to the crystal orientation, a well-ordered atomic column of Si is highly contrasted at the core of each NC in the ABF image. Interestingly, the surfaces of the NCs are also apparently contrasted with the background (i.e., carbon film), unlike the HR-TEM image in Fig. 1. From the ABF image, we cannot comprehensively elucidate the surface configuration of ncSi:H. To reveal an atomic-resolution structure of ncSi:H surface, we used the HAADF STEM technique. In Fig. 4d, we show an HAADF STEM image with a contrast that is proportional to the atomic number Z of the elements in each atomic column, allowing direct image interpretation. The brighter spots indicate atomic Si columns. In the present case, the HAADF imaging seems to give an image contrast that is higher than that of ABF imaging. Figure 4e shows a representative high-magnification HAADF STEM image of ncSi:H treated with a low-pass filter for noise reduction. The NC has a periodic arrangement of the atomic columns across the entire core of ~2 nm in diameter. The estimated *d*-spacing of 1.9 Å at the core corresponds very closely with that of the (220) plane of a diamond cubic crystalline Si. Interestingly, the *d*-spacing seems to increase in small steps from the core toward the surface. For example, as indicated in the image, the measured *d*-spacing half-way between the core and surface is 2.3 Å, which is 17% larger than the expected value, indicating the presence of a distorted diamond cubic structure in which an extended interatomic distance of Si-Si is observed. A partial lack of periodicity of the atomic columns can be seen everywhere near the surface. Furthermore, close to the surface, we see an amorphous configuration of atomic columns rather than a distorted lattice configuration. Such amorphous configurations including the heavily distorted structures of crystalline lattice occupying a broad range of the NC interior would allow different average distances between Si atoms, create defects caused by breaking Si-Si bonds, and produce strained Si lattice. Therefore, the phonon energy must be influenced by the disordered atoms, leading to the Raman spectral shift and broadening (see Fig. 4b)^61^. A Raman shift of 507 cm^−1^ would appear because of a smaller core size than that of ncSi-OD, being consistent with the characterization results of XRD and ATR-FTIR. Comparing the contrasted Raman spectra between ncSi-OD and ncSi:H, it is interesting to note that the alkyl monolayers possibly work to suppress the distortion of a diamond cubic Si structure.

Hannah, *et al*. report the role of a-Si as a nonradiative channel^67^. In their study, four sets of ncSi with different crystallinity were prepared by a radio frequency (RF) plasma method to compare time-resolved PL spectra recorded on a streak camera. Their comparison reveals that a PL decay feature of a few multiple of 10 ps arises from a nonradiative process associated with the amorphous structures. Part of their argument leads us to a possible origin for the nonradiative *e-h* recombination in the interior of the ncSi:H because of the amorphized layer at the ncSi:H surface. The role of a few-angstrom-thick structurally disordered surface layer on the PL properties of ncGe has also been discussed^65^. Although further studies are needed, our results obtained from Raman spectroscopic and HAADF-STEM observations suggest that the structurally disordered regions, including the amorphous phase, probably work as one of the nonradiative channels. In contrast, ncSi-OD is mainly composed of a diamond cubic lattice with a coherent structure and without an amorphous phase. Judging from the notable discrepancy in surface configuration between ncSi:H and ncSi-OD, the passivation by the monolayers possibly restrains the surface atoms from amorphization. Such a restraint effect based on the ligand chemistry contributes to the lack of DOS within the fundamental energy-gap, yielding the enhancement of the radiative relaxation process for recombination of photogenerated carriers. It is interesting to note that alkyl monolayers could behave as an anchor that suppresses the undesired surface reconstruction. A question arises as to why the NC surface of ncSi-OD is not distorted. A possible mechanism is the replacement of Si-H bonds by Si-OD before surface reconstruction occurs because the hydrogen-terminated NC surface takes time to be amorphized.

In the future, we would like to address the possibility for further enhancement of PL QYs. The present work exhibits a small decline of PL intensity with increasing temperature, which suggests the presence of thermally activated nonradiative trapping states in ncSi-OD. If such nonradiative channels became inactive even at high temperatures, the PL intensity would remain constant over the range from cryogenic to room temperatures^38^. According to that scenario, a dramatic enhancement in PL QY would rely upon surface passivation with shells that inhibit the creation of nonradiative relaxation channels, including the amorphous configuration that is caused by surface reconstruction.

## Conclusions

In summary, we investigated the PL properties and decay dynamics of freestanding Si NCs with different terminal groups, hydrogen atoms or alkyl monolayers, to ascertain the mechanism by which PL QYs are enhanced by hydrosilylation of 1-alkenes. The temperature dependences of the PL properties and decay dynamics were measured in the range 3–298 K. The evolution of PL intensity as a function of temperature implied the presence of a larger number of nonradiative channels in the interior of ncSi:H than in that of ncSi-OD. We showed for the first time that the temperature dependence of the bandgap energies of nanostructured Si could be reproduced by the modified Varshni relation as well as QDs of other semiconductors by using freestanding red-light-emitting ncSi:H as a model. Combinational analysis of the evolution of bandgap energy with PL decay dynamics as a function of temperature suggested that the confinement of photogenerated *e-h* carriers in ncSi is strengthened by termination with alkyl monolayers. Combining ABF and HAADF STEM imaging with Raman spectroscopy demonstrated that the ncSi:H surface layer is significantly distorted under structural reconstruction to generate an amorphous phase, whereas the passivation with alkyl monolayers suppresses the surface reconstruction to hold a bulk-like geometry (i.e., a diamond cubic lattice structure) in a broad range from the center toward the near-surface in ncSi. Such a structurally disordered region possibly works as a nonradiative relaxation channel to give a low PL QY. Interestingly, the passivation with alkyl monolayers works to prevent any undesired surface reconstruction, leading to the high PL QY.

## Methods

### Materials

Triethoxysilane (TES), 1-octadecene, and mesitylene were obtained from Sigma Aldrich, and used as received. All other chemicals were purchased from Wako Pure Chemical Industries Ltd. Japan and used as received. Water was purified and deionized using a Sartorius (arium 611 UV) water purification system.

### Preparation of ncSi embedded in SiO_2_

Both ncSi:H and ncSi-OD samples employed in this work were prepared according to the disproportionation of (HSiO_1.5_)_*n*_ at high temperature as reported in our previous paper^1^. In a typical synthesis, 5 mL of TES (6.0 g, 45 mmol) was added to a round-bottom flask equipped with a magnetic stir bar and stirred in Ar atmosphere using standard Schlenk techniques. 1.59 mL of HCl solution (water:HCl = 3:1 of volumetric percentage) was added drop wise slowly to the TES placed at the bottom of flask. A xerogel obtained was dried in vacuum for overnight. Next the dried gel placed in a quartz crucible was annealed at 1100 °C for 1 hr in vacuum conditions. After cooling to room temperature, the resulting dark brown solid products were mechanically ground in a mortar and pestle to yield fine, free-flowing powders.

### Preparation of ncSi:H liberated from SiO_2_

300 mg of the free-flowing powder was stirred for 60 min in alcoholic acid solution of 10 mL ethanol and 20 mL HF (48%) to liberate NCs from the oxide matrix. At the same time, the most of the outermost Si atoms were terminated with hydrogen atoms. The ncSi:H was centrifuged at a rotation speed of 16000 rpm, and washed with acetonitrile and dichloromethane. The precipitated product was dried overnight under vacuum.

### Preparation of ncSi-OD

During the preparation ncSi-OD we took special caution to avoid exposure of the surface in ambient atmosphere. In a first step for hydrosilylation of 1-octadecene, the ncSi:H soaked in a very small amount of dichloromethane was transferred to another Schlenk flask filled with argon gas without exposure to the air. A 5 mL mesitylene with 5 mL 1-octadecene was previously poured into the flask. Next, the mixture was degassed for at least 1 h with Ar bubbling. Hydrosilylation of 1-octadecene was carried out at 150 °C overnight in Ar atmosphere. The mixture was cooled to room temperature. A 30 mL ethanol was added to the mixture. Centrifugation at 16,000 rpm for 20 min was performed to wash the product, and the colourless supernatant was discarded. For the washing, we employed a mixture of toluene and ethanol (1:3, volumetric ratio), and the solution was subjected to centrifugation several times to remove mesitylene and unreacted 1-octadecene. Finally the precipitated product was dried overnight under vacuum.

### Characterization

X-ray powder diffraction (HT-XRD, RINT-TTR II and Reactor X, Rigaku, Japan) was used to characterize the major crystalline phases of the samples, and to estimate the diameters of the samples from the diffraction linewidths of (111) phase using Scherrer equation. For the TEM and STEM observation, the sample was prepared by drop-casting the dilute dispersion of ncSi:H in chloroform onto the copper grid covered with ultrathin amorphous carbon film. A low magnification image was acquired to characterize the size and morphology of the ncSi:H using a HR-TEM, JEOL JEM-2100 operated at 200 kV. The sample was also observed on the JEOL JEM-ARM200F with STEM mode operating at 200 kV for the detailed observation at high magnification. This offers an unprecedented opportunity to probe structures with sub-Angström resolution. In the STEM observation, the bright-field and the dark-field images were acquired with in-line and HAADF detectors, respectively. A low-pass filter was applied to the image for noise reduction. Attenuated total reflectance Fourier transform infrared spectroscopy (ATR-FTIR) measurements were carried out in the frequency range of 750–4000 cm^−1^ using a germanium prism on a JASCO FTIR 4100 spectrometer. Raman measurements were carried out on NC films deposited from dichloromethane colloids on a gold-coated substrate. Raman spectra at a single excitation wavelength (532 nm) were on a slit-scanning Raman microscope (RAMAN-11; Nanophoton, Osaka, Japan). The steady-state absorption spectra of the sample dispersed in non-luminescent dichloromethane were recorded with a JASCO V-650 spectrophotometer. PL and PLE measurements were carried out using a modular double grating Czerny–Turner monochromator and an iHR 320 emission monochromator (1200 lines/mm of gratings) coupled to an InGaAs Hamamatsu photodetector on a NanoLog Horiba Jovin Yvon spectrofluorometer with 450 W xenon arc lamp. The spectral resolution of the system is around 0.3 nm. The time-resolved PL decay curves were acquired with the same NanoLog Horiba Jovin Yvon spectrofluorometer, using a pulsed spectral LED of 370 nm emission. The absolute PL quantum yields (PL QYs) were measured at room temperature using the QY measurement system C9920–02 from Hamamatsu Photonics Co. Ltd with a 150 W xenon lamp coupled to a monochromator for wavelength discrimination, an integrating sphere as a sample chamber, and a multichannel analyzer for signal detection. The ncSi:H and the ncSi-OD in powder form were employed as samples for the estimation of PL QYs. For the temperature-dependent PL measurements, the samples were placed into an Opticool Stage cryostat connected to GM cooler and controlled by MercuryiTC temperature controller, which allowed tuning the sample temperature in the range from 3 to 298 K. The samples for temperature-dependent fluorescence study were prepared by casting a non-luminescent dichloromethane solution of the NCs over the surface of quartz glass substrate followed by drying under vacuum conditions.

## Additional Information

**How to cite this article**: Ghosh, B. *et al*. Origin of the Photoluminescence Quantum Yields Enhanced by Alkane-Termination of Freestanding Silicon Nanocrystals: Temperature-Dependence of Optical Properties. *Sci. Rep*. **6**, 36951; doi: 10.1038/srep36951 (2016).

**Publisher’s note:** Springer Nature remains neutral with regard to jurisdictional claims in published maps and institutional affiliations.
